# Suaveolic Acid: A Potent Phytotoxic Substance of *Hyptis suaveolens*

**DOI:** 10.1155/2014/425942

**Published:** 2014-10-21

**Authors:** A. K. M. Mominul Islam, Osamu Ohno, Kiyotake Suenaga, Hisashi Kato-Noguchi

**Affiliations:** ^1^Department of Applied Biological Science, Faculty of Agriculture, Kagawa University, 2393 Ikenobe, Miki, Kagawa 761-0795, Japan; ^2^Department of Chemistry, Faculty of Science and Technology, Keio University, 3-14-1 Hiyoshi, Kohoku, Yokohama 223-8522, Japan

## Abstract

*Hyptis suaveolens* (Lamiaceae) is an exotic invasive plant in many countries. Earlier studies reported that the aqueous, methanol, and aqueous methanol extract of *H. suaveolens* and its residues have phytotoxic properties. However, to date, the phytotoxic substances of this plant have not been reported. Therefore, the objectives of this study were isolation and identification of phytotoxic substances of *H. suaveolens*. Aqueous methanol extract of this plant was purified by several chromatographic runs through bioassay guided fractionation using garden cress (*Lepidium sativum*) as a test plant. Final purification of a phytotoxic substance was achieved by reverse phase HPLC and characterized as 14*α*-hydroxy-13*β*-abiet-8-en-18-oic acid (suaveolic acid) by high-resolution ESI-MS, ^1^H-,^13^C-NMR, CD, and specific rotation. Suaveolic acid inhibited the shoot growth of garden cress, lettuce (*Lactuca sativa*), Italian ryegrass (*Lolium multiflorum*), and barnyard grass (*Echinochloa crus-galli*) at concentrations greater than 30 *µ*M. Root growth of all but lettuce was also inhibited at concentrations greater than 30 *µ*M. The inhibitory activities were concentration dependent. Concentrations required for 50% growth inhibition of suaveolic acid for those test plant species were ranged from 76 to 1155 *µ*M. Therefore, suaveolic acid is phytotoxic and may be responsible for the phytotoxicity of *H. suaveolens* plant extracts.

## 1. Introduction

*Hyptis suaveolens* belonging to Lamiaceae family is a soft suffrutescent and ruderal weed that normally grows along the roadsides and the wet margins of ponds. The plant is native to tropical America but now distributed throughout the whole world from tropical to subtropical regions and, therefore, the plant is sometimes regarded as pantropical weed [1, 2].* H. suaveolens *can reach 2 m in height, forms dense clumps, and is considered as one of the world's most noxious exotic invasive species invading the natural ecosystems including savannah at an alarming rate [3, 4]. Besides the hurtful effect of* H. suaveolens* on natural ecosystems as invader weed, the plant is an important source of many pharmacological and industrial constituents that have been reviewed by Nayak et al. [5], Barbosa et al. [6], and Sharma et al. [7]. But its utilization for the pharmacological or industrial purpose is now much lower than its damage caused to the adjoining areas [8].

It has also been reported that the growth and establishment of other plant species near their clumps are quite restricted [8], but the specific reasons that lead to the dominance of* H. suaveolens *still remain unclear. One of the plausible reasons for such interference could be due to the phytotoxicity of this plant. However, to the best of our knowledge, there is no study that has been reported to address the phytotoxic substances of* H. suaveolens*, except few preliminary phytotoxicity studies on the germination and growth of different test plant species [9–13].

We previously reported the phytotoxic activities of aqueous methanol extract of* H. suaveolens* on the germination and seedling growth of several weed and crop species [13]. Therefore, current research was envisaged with the aim of isolation and identification of its phytotoxic substances from the aqueous methanol extract of* H. suaveolens*. The findings of this research will be helpful to understand the plant-plant interaction of* H. suaveolens* under natural settings.

## 2. Materials and Methods

### 2.1. Plant Materials

Whole plants (leaves, stems, and roots) of* Hyptis suaveolens* (L.) Poit. were collected from Bangabandhu Sheikh Mujibur Rahman Agricultural University, Gazipur-1706, Bangladesh, during July-August, 2013. The plants were then washed with tap water; dried under sunlight; and kept in a refrigerator at 2°C until extraction.

### 2.2. Test Plant Species

Two dicotyledonous species, garden cress (*Lepidium sativum* L.) and lettuce (*Lactuca sativa* L.), and two monocotyledonous, Italian ryegrass (*Lolium multiflorum* L.) and barnyard grass (*Echinochloa crus-galli* L. Beauv.), were used for the bioassays as test plant species. The dicotyledonous species were selected for their known seedling growth and higher sensitivity to phytotoxic substances [14], and monocotyledonous were selected for their common abundance in the farmers' fields throughout the world. Garden cress seeds were obtained from Nakahara Seed Product Co. Ltd. (Fukuoka, Japan), and seeds of lettuce and Italian ryegrass were obtained from Takii Co. Ltd. (Kyoto, Japan). The barnyard grass seeds were collected from the farmer's fields (Kagawa, Japan).

### 2.3. Extraction and Extract Separation

Whole plants (leaves, stems, and roots) of dried* H. suaveolens* were cut into small pieces, grinded into powder by a mechanical grinder, and extracted with 300 mL of 70% (v/v) aqueous methanol for 48 h. Filtration was carried out by one layer of filter paper (number 2; Toyo Ltd., Japan). The residue was then extracted again with 300 mL of methanol for 48 h and filtered. Two filtrates were combined and evaporated to dryness at 40°C to produce an aqueous residue. The residue was then adjusted to pH 7.0 with 1 M phosphate buffer and partitioned three times against an equal volume of ethyl acetate to obtain aqueous and ethyl acetate fractions. The biological activities of both fractions were determined by garden cress growth bioassay due to its higher sensitivity to phytotoxic substances.

### 2.4. Bioassay of Separated Fractions

A portion of aqueous or ethyl acetate fractions were dissolved in methanol and added to a sheet of filter paper (number 2) in 28 mm Petri dishes. The final assay concentrations were 10, 30, and 100 mg dry weight equivalent extract mL^−1^. After evaporating the methanol in a draft chamber, the filter paper was moistened with 0.6 mL of 0.05% (v/v) aqueous solution of nontoxic surfactant: polyoxyethylene sorbitan monolaurate (Tween 20; Nacalai, Japan). Ten seeds of garden cress were sown in the Petri dishes. The shoot and root lengths of the seedlings were calculated 48 h after incubation in darkness at 25°C. Control Petri dishes were also maintained in each experiment using only Tween 20 without plant extracts. The percentage length of seedlings was then measured by reference to the length of control. The sample preparation and adding to Petri dishes were done separately for each fraction of the extract.

### 2.5. Purification of Active Substance

The ethyl acetate fraction was evaporated to dryness after drying over anhydrous Na_2_SO_4_. The crude material was then separated by silica gel column (60 g of silica gel 60, spherical, 70–230 mesh, Nacalai), eluted stepwise with *n*-hexane containing increasing amounts of ethyl acetate (10% per step, v/v), ethyl acetate, acetone, and methanol (150 mL per step). The biological activity of all the collected fractions was determined using garden cress bioassay according to the aforesaid procedure, and inhibitory activity was found in the fraction obtained with 60% *n*-hexane in ethyl acetate. After evaporation, the active residue was applied to Sephadex LH 20 (50 g, GE Healthcare Bio-Sciences AB, Sweden) and eluted with 20, 30, 40, 50, 60, 70, 80, and 90% (v/v) of aqueous methanol and methanol (150 mL per step). The most active fraction eluted with 70% aqueous methanol was dissolved in 20% (v/v) aqueous methanol (1.0 mL) and loaded onto reverse-phase C_18_ cartridges (YMC Co. Ltd., Japan). The cartridge was eluted with 20, 30, 40, 50, 60, 70, 80, and 90% (v/v) aqueous methanol and methanol (30 mL per step). The active fraction obtained from 70% aqueous methanol was finally purified by reverse phase HPLC (500 × 10 mm I.D., ODS AQ-325, YMC) eluted at a flow rate of 1.5 mL min^−1^ with 80% (v/v) aqueous methanol and detected at 220 nm. The strongest inhibitory activity was found in a peak fraction eluted between 126 and 139 min as a colourless substance. This substance was then characterized by high resolution ESI-MS, ^1^H-, ^13^C-NMR, CD, and specific rotation (see Supplementary Figures S1–S7 in Supplementary Material available online at http://dx.doi.org/10.1155/2014/425942).

### 2.6. Bioassay of Suaveolic Acid

Suaveolic acid was dissolved in 0.2 mL of methanol to prepare assay concentrations and added to a sheet of filter paper (number 2) in 28 mm Petri dishes. Ten seeds of garden cress or lettuce or 10 seedlings of Italian ryegrass or barnyard grass (germinated in dark for 24–36 h at 25°C) were sown in the Petri dishes and biological activities were determined according to the same protocol mentioned above.

### 2.7. Statistical Analysis

The bioassays were conducted with three replications and repeated twice using a completely randomized design (CRD) with 10 seeds or seedlings for each determination. Student's *t*-test was performed to examine the significant differences between treatment and controls. The concentration required for 50% growth inhibition (*I*_50_) of suaveolic acid for the test plant species was determined by a logistic regression equation of the concentration response curves.

## 3. Results and Discussion

### 3.1. Isolation and Structure Determination of the Phytotoxic Substance

**Figure 1:**
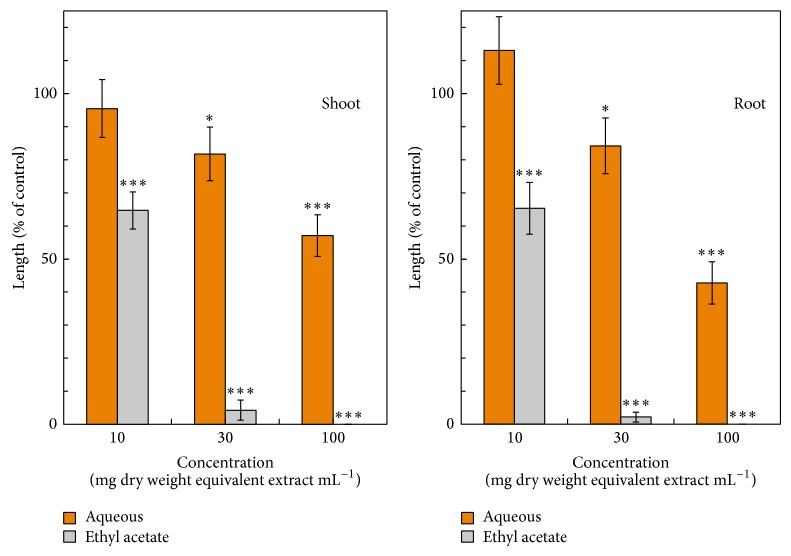
Effects of ethyl acetate and aqueous fractions isolated from the aqueous methanol extract of* H. suaveolens* on shoot and root growth of garden cress. Concentrations of tested samples corresponded to the extracts obtained from 10, 30, and 100 mg dry weight of* H. suaveolens*. Vertical bars represent error bars with standard deviations. Means ± SE from three independent experiments with 10 seeds for each determination are shown. Asterisks indicate a significant difference between control and treatment ^*^ *P* < 0.05 and ^***^ *P* < 0.001.

The ethyl acetate fraction of the aqueous methanol extract at 100 mg dry weight equivalent extract mL^−1^ completely inhibited the shoot and root growth of garden cress. At the same concentration, the aqueous fraction of the extract inhibited the shoot and root growth by 57 and 42% of control, respectively (Figure 1). Though both fractions showed inhibitory activity on the shoot and root growth of garden cress, the ethyl acetate fraction was more phytotoxic than the aqueous fraction. Therefore, the isolation of active substances was further continued with ethyl acetate fraction.

**Figure 2:**
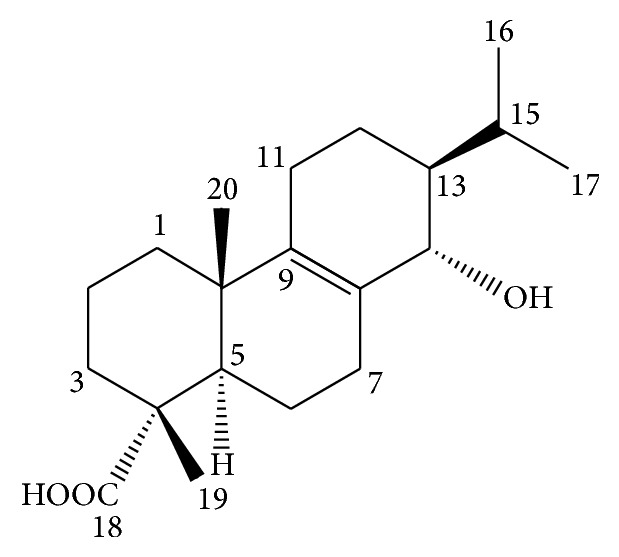
Chemical structure of suaveolic acid.

The ethyl acetate fraction was then purified by silica gel column, Sephadex LH 20 column, reverse phase C_18_ cartridges, and HPLC (ODS, MeOH-H_2_O), and a phytotoxic substance was isolated through bioassay guided fractionation method using garden cress as a test plant. This phytotoxic substance was further characterized by high resolution ESI-MS, ^1^H-, ^13^C-NMR, CD, and specific rotation (supplementary materials S1–S7). The molecular formula of the isolated compound was C_20_H_32_O_3_ by high resolution ESI-MS [*m/z* 319.2232 (M–H)^−^; calcd for C_20_H_31_O_3_, 319.2273, Δ = −4.1 mmu]. The ^1^H NMR (400 MHz, CD_3_OD) spectrum of the compound showed *δ*_H_: 3.73 (brd,* J* = 8.8 Hz, 1 H, H14), 2.44 (m, 1 H, H7b), 2.06 (m, 1 H, H11b), 2.04 (m, 1 H, H5), 2.04 (m, 1 H, H15), 1.89 (m, 1 H, H11a), 1.86 (m, 1 H, H7a), 1.82 (m, 1 H, H3b), 1.78 (m, 1 H, H1b), 1.69 (m, 1 H, H12b), 1.68 (m, 1 H, H2b), 1.59 (m, 1 H, H6b), 1.58 (m, 1 H, H2a), 1.56 (m, 1 H, H3a), 1.38 (brdd,* J* = 7.4, 12.1 Hz, 1 H, H6a), 1.31 (m, 1 H, H13), 1.18 (s, 3 H, H19), 1.16 (m, 1 H, H1a), 1.08 (brdd,* J* = 4.3, 12.1 Hz, 1 H, H12a), 1.02 (s, 3 H, H20), 0.96 (d,* J* = 6.7 Hz, 3 H, H17), and 0.81 (d,* J* = 7.0 Hz, 3 H, H16). The ^1^H NMR (400 MHz, CDCl_3_) spectrum showed *δ*_H_: 3.81 (brd,* J* = 8.1 Hz, 1 H, H14), 2.42 (m, 1 H, H7b), 1.26 (m, 1 H, H13), 1.19 (s, 3 H, H19), 1.08 (brdd,* J* = 3.8, 11.4 Hz, 1 H, H12a), 0.98 (s, 3 H, H20), 0.95 (d,* J *= 7.0 Hz, 3 H, H17), and 0.81 (d,* J* = 6.7 Hz, 3 H, H16). The ^13^C NMR (100 MHz, CD_3_OD) spectrum of the compound showed *δ*_C_: 184.0 (C18), 142.9 (C9), 130.7 (C8), 73.8 (C14), 49.4 (C13), 49.2 (C4), 47.5 (C5), 38.3 (C10), 38.0 (C3), 36.8 (C1), 29.2 (C7), 28.0 (C15), 25.3 (C11), 22.7 (C12), 22.4 (C6), 21.7 (C17), 19.6 (C20), 19.3 (C2), 17.2 (C16), and 17.2 (C19). The circular dichroism (CD) spectrum of this compound in MeOH showed a negative cotton effect below 215 nm (*λ* 200 nm, Δ*ε* − 5.8). The specific rotation of the compound ([*α*]_D_^25^) was measured to be +46.6° (*c* 0.1, MeOH). From the comparison of these data with literatures [15, 16], this compound was determined as an abietane type diterpenes, 14*α*-hydroxy-13*β*-abiet-8-en-18-oic acid (suaveolic acid) (Figure 2).

### 3.2. Phytotoxic Activity of Suaveolic Acid

**Figure 3:**
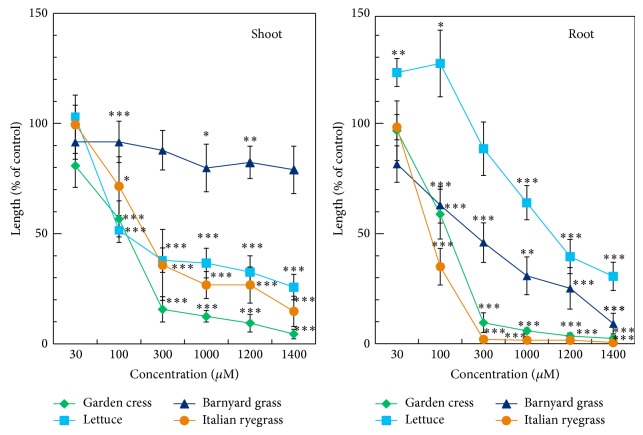
Effects of suaveolic acid on the shoot and root growth of garden cress, lettuce, Italian ryegrass, and barnyard grass seedlings. Means ± SE from three independent experiments with 10 seedlings for each determination are shown. Asterisks indicate a significant difference between control and treatment. ^*^ *P* < 0.05, ^**^ *P* < 0.01, and ^***^ *P* < 0.001.

Suaveolic acid at concentrations greater than 30 *μ*M inhibited the shoot and root growth of garden cress, Italian ryegrass, and barnyard grass and the shoot growth of lettuce (Figure 3). The lettuce roots were inhibited at concentrations greater than 100 *μ*M (Figure 3). The inhibitory activities were concentrations dependent. The *I*_50_ values of suaveolic acid for the shoot and root growth of those test plant species were ranged from 100 to 236 and 76 to 1155 *μ*M, respectively (Table 1). The exogenous concentration of suaveolic acid is at least 406 *μ*mol kg^−1^, as 7.4 mg of the substance (MW 320) was isolated from 57 g dry weight of* H. suaveolens*.

The shoot growth of dicotyledonous species (garden cress and lettuce) was more sensitive to suaveolic acid than their roots, whereas monocotyledonous species (barnyard grass and Italian ryegrass) showed the opposite (Table 1). Similar results of inhibition of those test plant species were also obtained in our preliminary experiments with the aqueous methanol extract of* H. suaveolens* [13]. These results indicated the involvement of suaveolic acid on the phytotoxic activity of* H. suaveolens* plant extract.

The concentrations lower than the threshold for inhibition of suaveolic acid have tendency to stimulate the root growth of lettuce (Figure 3). It has been reported that phytotoxic compound can stimulate the seedling growth at very low concentrations but can inhibit the same at higher concentrations [17–20]. This phenomenon is known as hormesis [21–23].

Although the presence of suaveolic acid in the aerial parts of* H. suaveolens* has been reported 40 years before [15], very fews are known about its biological activity. Prawatsri et al. [16] isolated few abietane diterpenes including suaveolic acid from the dried whole plant of* H. suaveolens* and reported that suaveolic acid has antimycobacterial properties. They observed no activities of suaveolic acid against human oral carcinoma, human breast cancer, and human lung cancer cells. Grassi et al. [24] reported the anti-inflammatory activity of methyl suaveolate, a synthesized product of suaveolic acid [15, 25]. However, there have been no reports about the phytotoxic activity of suaveolic acid. To the best of our knowledge, this is first report about the phytotoxic properties of suaveolic acid.

Under certain conditions phytotoxic substances may release from the phytotoxic plants and suppress the germination, growth, and establishment of neighbouring plants by affecting their physiological properties [26, 27] or indirectly by modifying the rhizosphere soil properties through influencing the microbial biomass carbon and microbial community [28–30]. Since* H. suaveolens* is an annual weed, suaveolic acid may possibly release into the surrounding environment through the decomposition of their aerial parts (leaf or stem) and accelerate the invasion or dominancy of* H. suaveolens* in their new range. However, this assumption clearly needs further investigations to understand the releasing mechanism of suaveolic acid into the environment and also its stability in the soil under field conditions.

## 4. Conclusion

A phytotoxic substance, suaveolic acid (14*α*-hydroxy-13*β*-abiet-8-en-18-oic acid), has been isolated from the aqueous methanol extract of* H. suaveolens*. At concentrations greater than 30 *μ*M, suaveolic acid showed phytotoxicity against the shoot and root growth of garden cress, Italian ryegrass, and barnyard grass and lettuce shoots. Roots of lettuce were inhibited at concentrations greater than 100 *μ*M. The *I*_50_ values for the seedling growth of the test plant species ranged from 76 to 1155 *μ*M. Therefore, suaveolic acid may be responsible for the phytotoxic activity of* H. suaveolens* plant extract. The findings of this research may be helpful to explore the interaction of* H. suaveolens* with their neighbouring plants species under natural settings.

## Supplementary Material

Figure S1: 1H NMR spectrum of suaveolic acid (400 MHz, CD3OD)
Figure S2: 1H NMR spectrum of suaveolic acid (400 MHz, CDCl3)
Figure S3: 13C NMR spectrum of suaveolic acid (100 MHz, CD3OD)
Figure S4: COSY spectrum of suaveolic acid (400 MHz, CD3OD)
Figure S5: HMQC spectrum of suaveolic acid (400 MHz, CD3OD)
Figure S6: HMBC spectrum of suaveolic acid (400 MHz, CD3OD)
Figure S7: CD spectrum of suaveolic acid (MeOH)

## Figures and Tables

**Table 1:** *I*_50_ values of suaveolic acid for shoot and root growth of the test plant species.

Test plant species	Shoot growth		Root growth	
	*I*_50_ (*μ*M)	Coefficient of correlation (*R* ^2^)	*I*_50_ (*μ*M)	Coefficient of correlation (*R* ^2^)
Garden cress	100.3	0.970	111.6	0.990
Lettuce	158.8	0.914	1155.2	0.847
Barnyard grass	Not converged	—	257.1	0.998
Italian ryegrass	235.6	0.878	75.8	1.00

The values were determined by a logistic regression analysis after bioassays.

## Abbreviations

$I_{50}$: Concentration required for 50% growth inhibition
HPLC: High performance liquid chromatography
HRESI-MS: High-resolution electrospray ionisation mass spectrometry
NMR: Nuclear magnetic resonance
CD: Circular dichroism.
