# Supplementary material for: Suaveolic Acid: A Potent Phytotoxic Substance of Hyptis suaveolens
Source: ScientificWorldJournal. 2014 Oct 21;2014:425942. doi: 10.1155/2014/425942 (PMC4227406; doi:10.1155/2014/425942)
Supplement: Supplementary file 1 — Figure S1: 1H NMR spectrum of suaveolic acid (400 MHz, CD3OD) Figure S2: 1H NMR spectrum of suaveolic acid (400 MHz, CDCl3) Figure S3: 13C NMR spectrum of suaveolic acid (100 MHz, CD3OD) Figure S4: COSY spectrum of suaveolic acid (400 MHz, CD3OD) Figure S5: HMQC spectrum of suaveolic acid (400 MHz, CD3OD) Figure S6: HMBC spectrum of suaveolic acid (400 MHz, CD3OD) Figure S7: CD spectrum of suaveolic acid (MeOH) [file 425942.f1.pdf]

List of Contents

Figure S1. ^{1}H NMR spectrum of suaveolic acid (400 MHz, CD_3OD)

Figure S2. ^{1}H NMR spectrum of suaveolic acid (400 MHz, $CDCl_3$)

Figure S3. ^{13}C NMR spectrum of suaveolic acid (100 MHz, CD_3OD)

Figure S4. COSY spectrum of suaveolic acid (400 MHz, CD_3OD)

Figure S5. HMQC spectrum of suaveolic acid (400 MHz, CD_3OD)

Figure S6. HMBC spectrum of suaveolic acid (400 MHz, CD_3OD)

Figure S7. CD spectrum of suaveolic acid (MeOH)

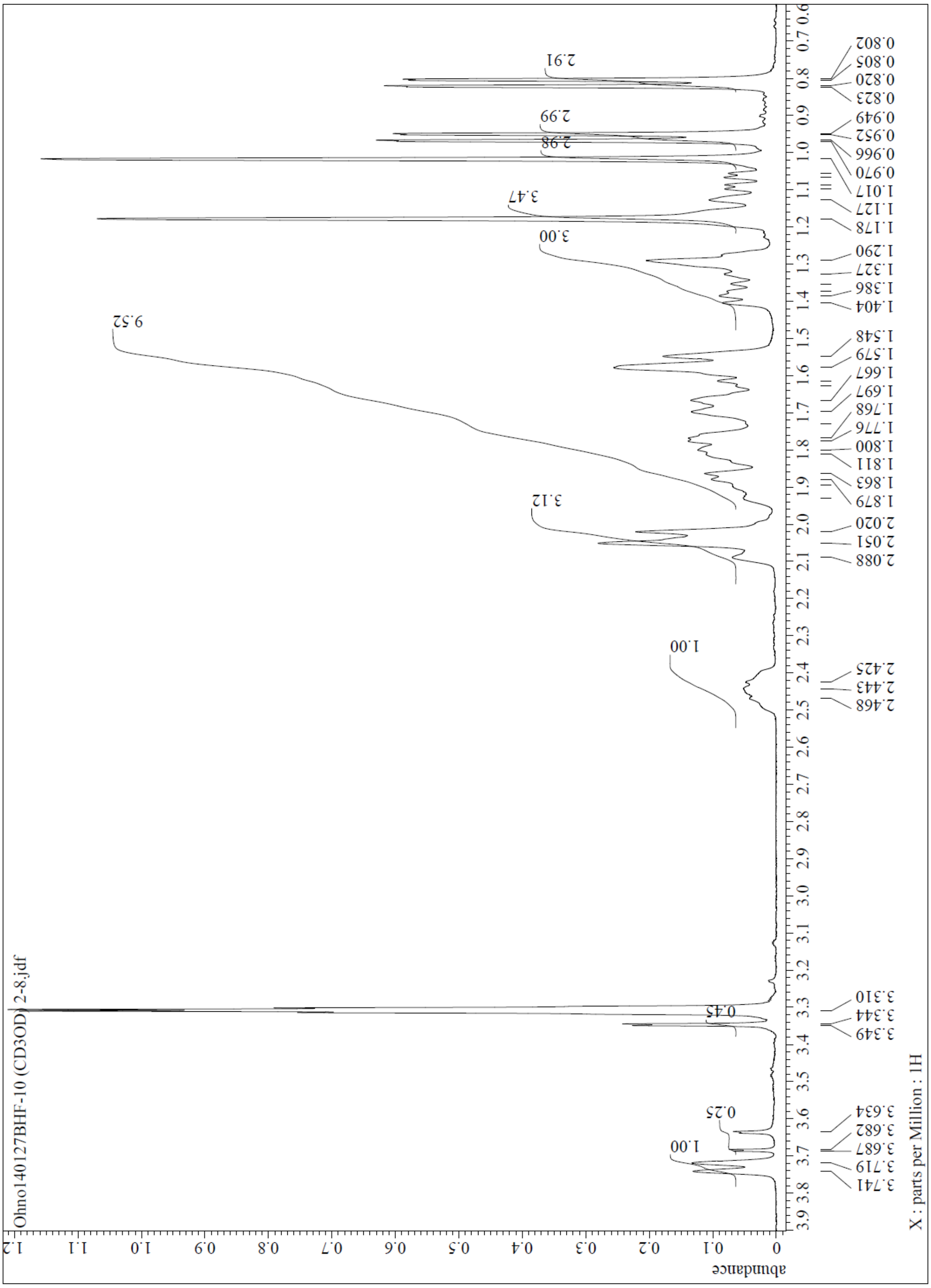

Figure S1. ^{1}H NMR spectrum of suaveolic acid (400 MHz, CD_3OD)

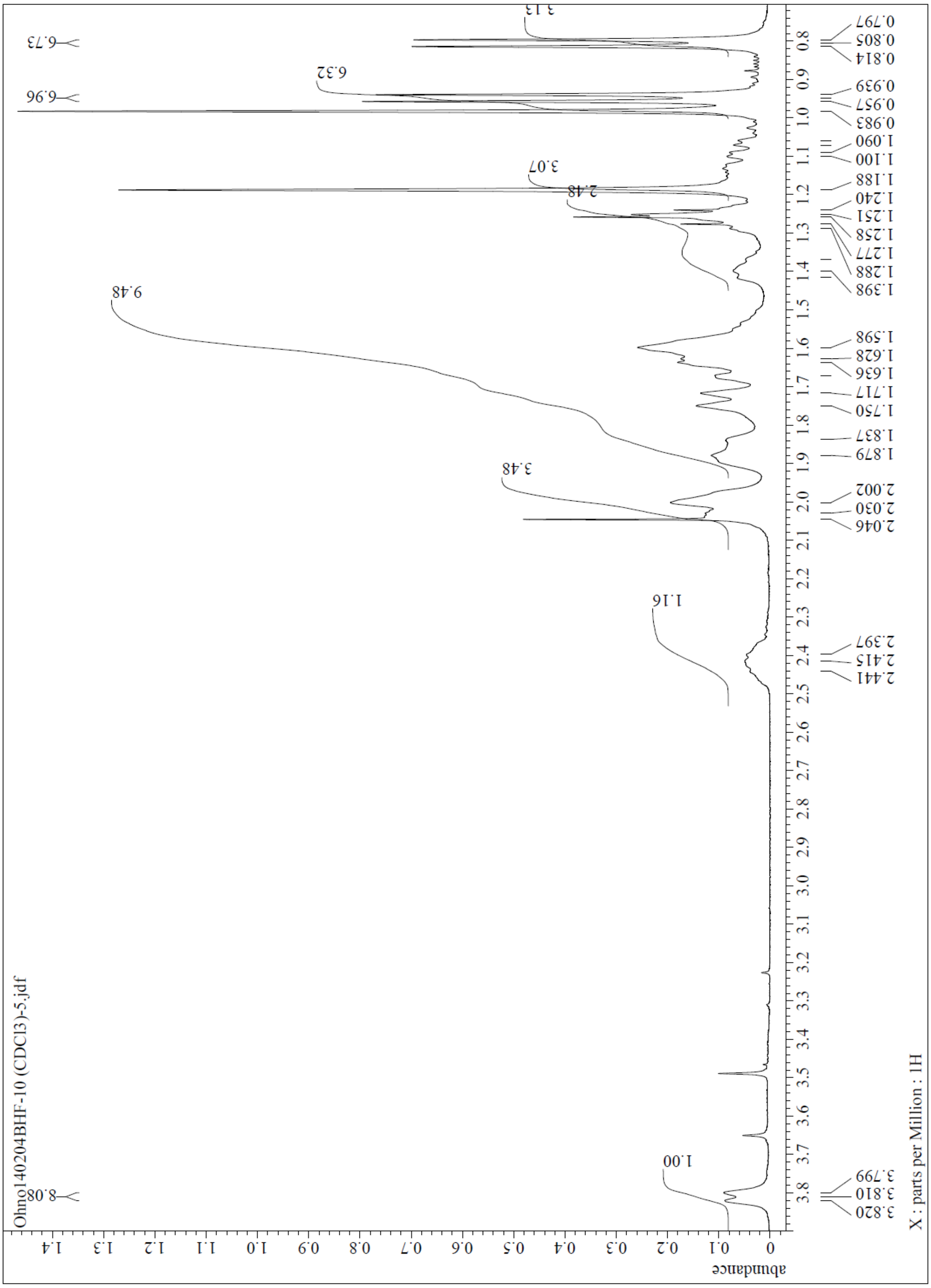

Figure S2. ^{1}H NMR spectrum of suaveolic acid (400 MHz, $CDCl_3$)

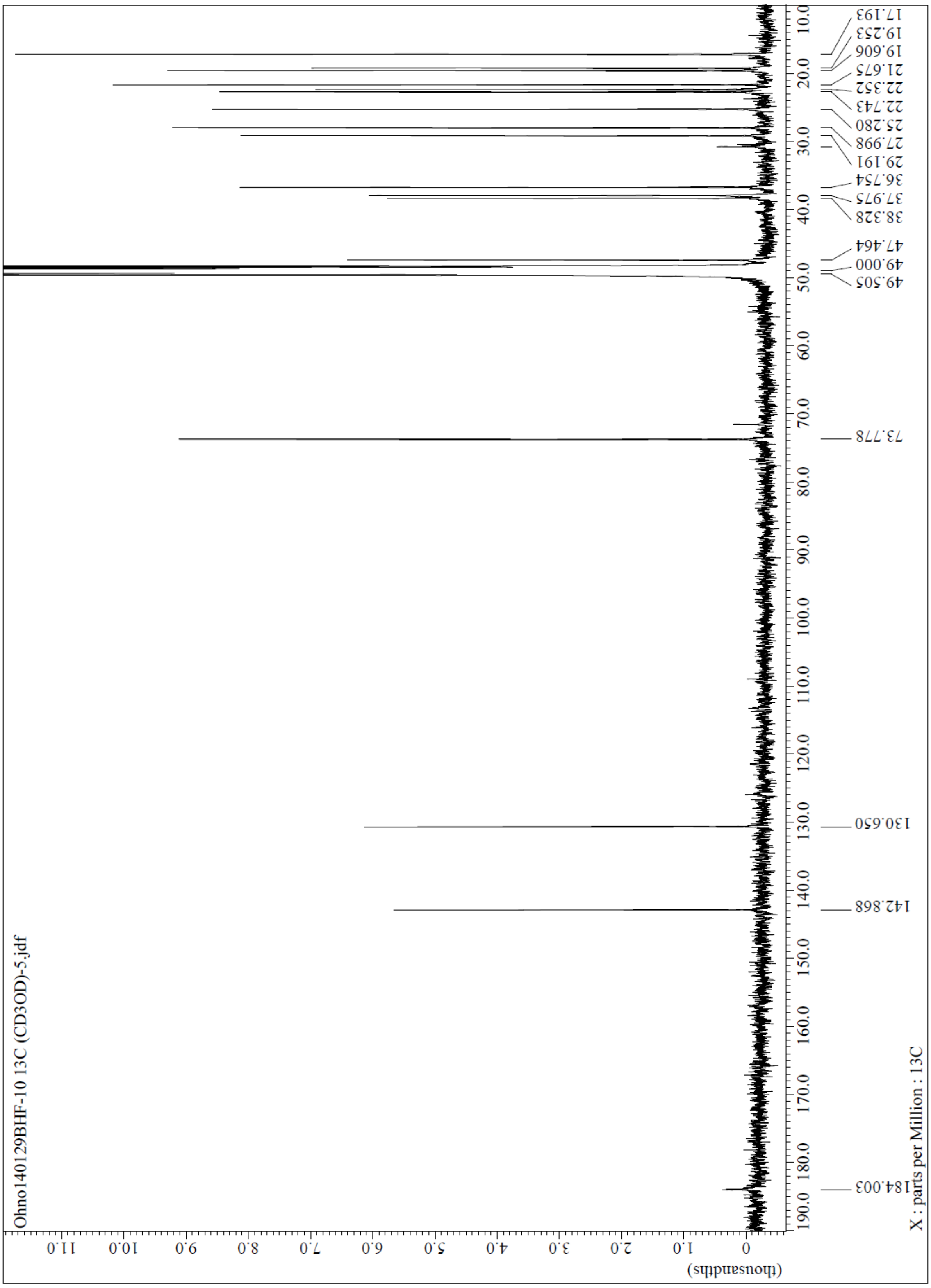

Figure S3. ^{13}C NMR spectrum of suaveolic acid (100 MHz, CD_3OD)

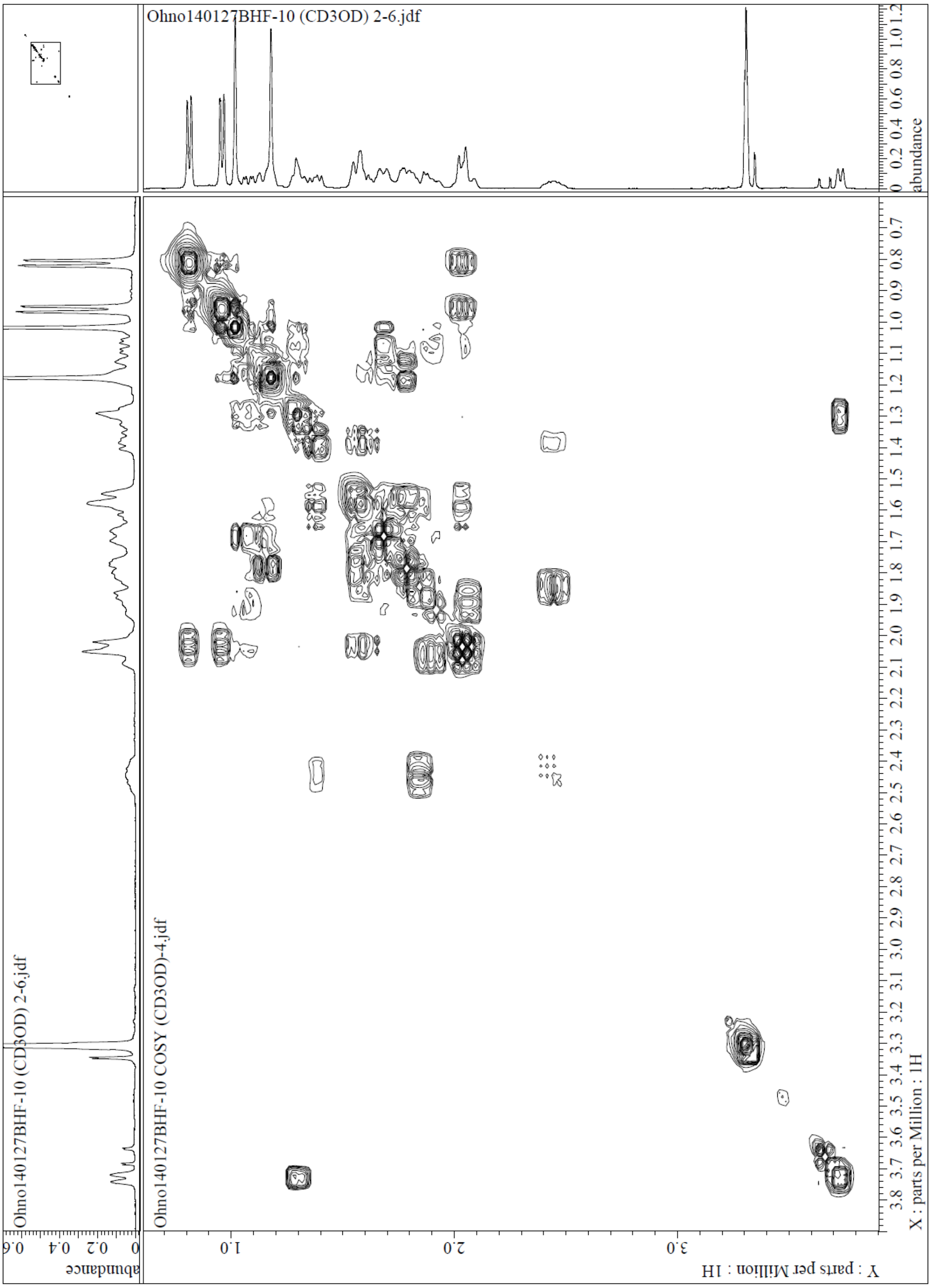

Figure S4. COSY spectrum of suaveolic acid (400 MHz, CD_3OD)

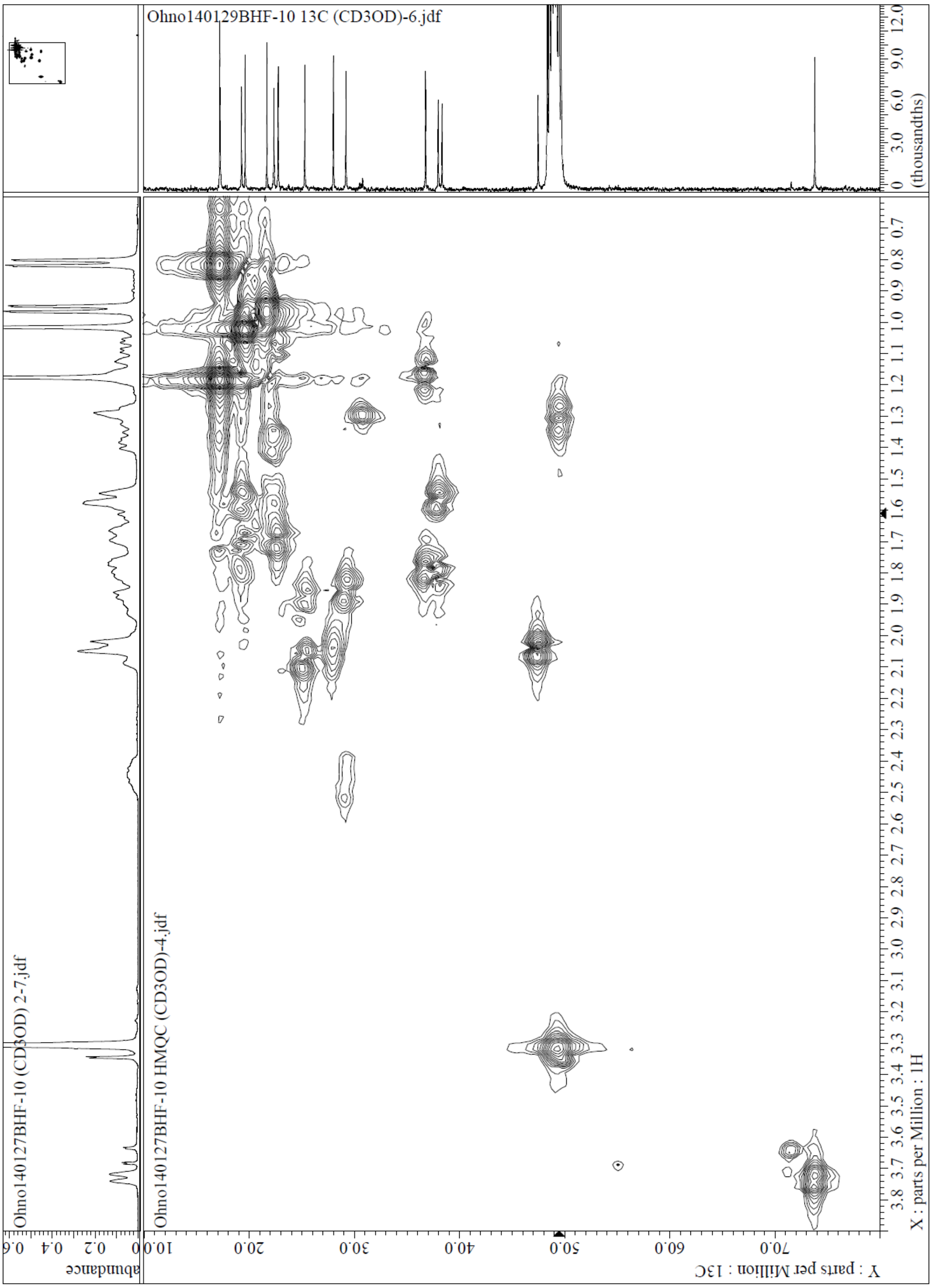

Figure S5. HMQC spectrum of suaveolic acid (400 MHz, CD_3OD)

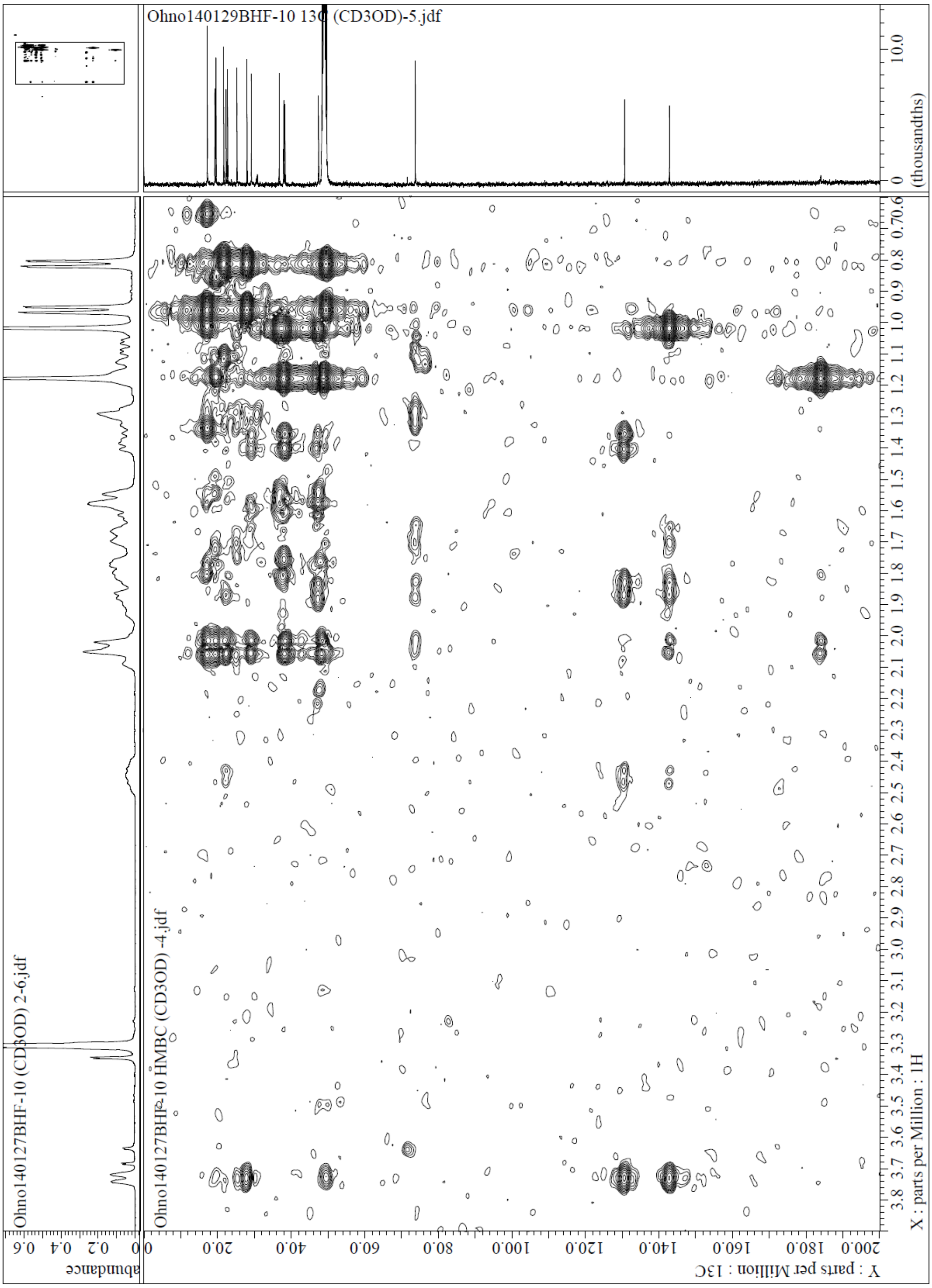

Figure S6. HMBC spectrum of suaveolic acid (400 MHz, CD_3OD)

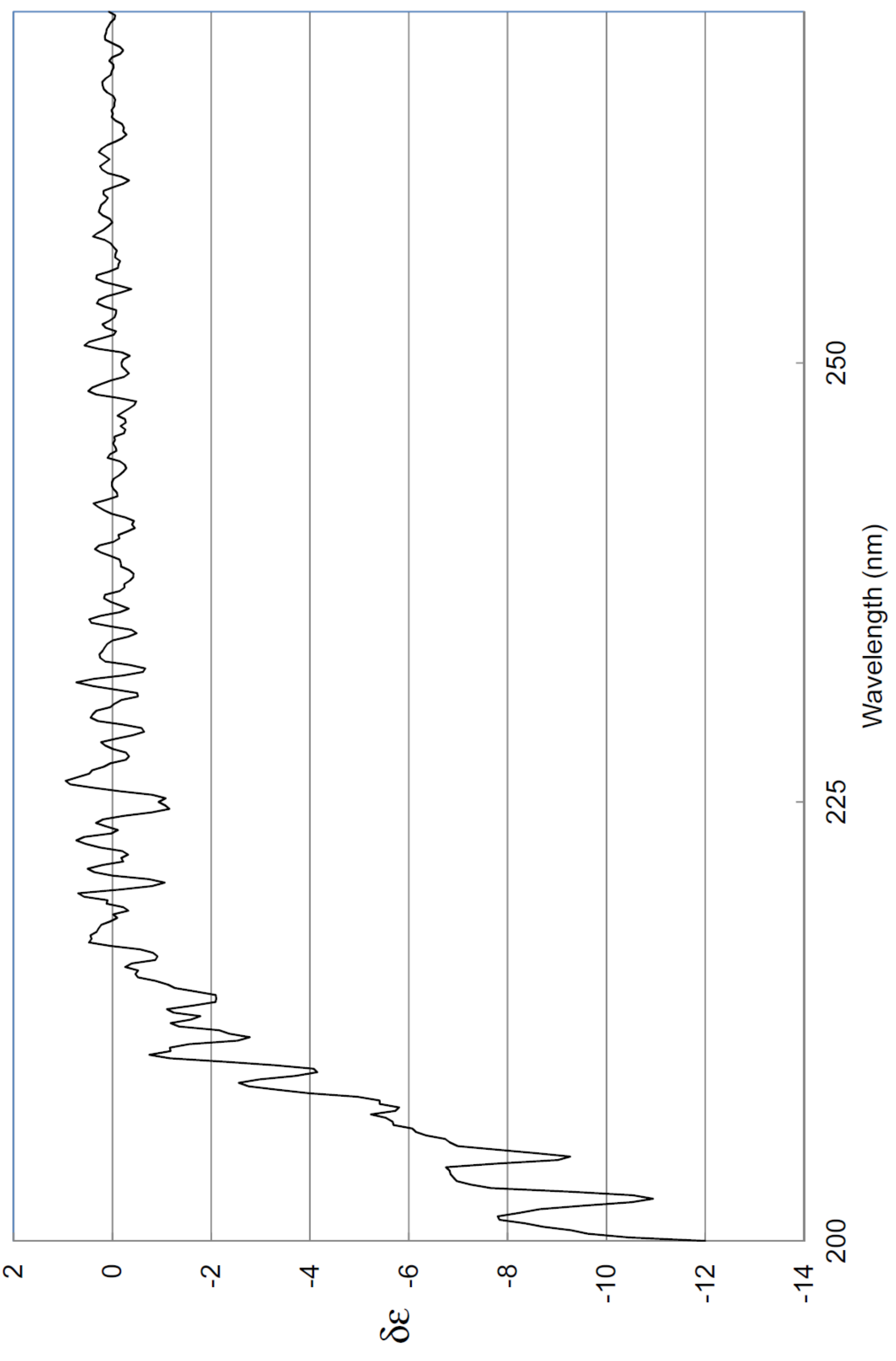

Figure S7. CD spectrum of suaveolic acid (MeOH)
